# Probiotics isolated from yaks improves the growth performance, antioxidant activity, and cytokines related to immunity and inflammation in mice

**DOI:** 10.1186/s12934-019-1161-6

**Published:** 2019-06-19

**Authors:** Aoyun Li, Yaping Wang, Zhixing Li, Hammad Qamar, Khalid Mehmood, Lihong Zhang, Juanjuan Liu, Hui Zhang, Jiakui Li

**Affiliations:** 1 College of Veterinary Medicine, Huazhong Agricultural University, Wuhan, 430070 People’s Republic of China; 2 University College of Veterinary & Animal Sciences, The Islamia University of Bahawalpur, Bahawalpur, 63100 Pakistan; 3 College of Animals Husbandry and Veterinary Medicine, Tibet Agricultural and Animal Husbandry University, Linzhi, 860000 Tibet People’s Republic of China

**Keywords:** Yaks, *Bacillus subtilis*, *Bacillus velezensis*, Digestive enzyme activity, Immunity

## Abstract

**Background:**

Yaks living in the high-altitude hypoxic environment of Tibetan plateau (3600 m) have special gut microbes. However, it is still little research on yak probiotics until now. Therefore, the purpose of our study was to evaluate the growth promoting effect, antioxidant capability, immune effect, and anti-inflammatory ability of *Bacillus subtilis* and *Bacillus velezensis* isolated from Tibetan yaks in mice model.

**Results:**

The results showed that the isolated strains supplementation not only improve the growth performance but also increased the length of villus in the small intestine and intestinal digestive enzyme activity. Importantly, we observed that the T-AOC, SOD, and GSH-PX levels were increased and the MDA content was reduced in probiotic-treated mice, which implied that probiotics supplementation can ameliorate the antioxidative activity of mice. The levels of AST and ALT correlated with the hepatic injury were reduced and the levels of AKP, TP, GLB, ALB, Ca, and P were markedly higher than those in the control group. Additionally, mice treated with probiotics exhibited higher serum IgG, IgM and IgA, which can reflect the immune status to some extent. At the same time, the major pro-inflammatory factor TNF-α, IL-6, and IL-8 were down-regulated and the anti-inflammatory factor IL-10 was up-regulated compared with the control groups.

**Conclusions:**

In conclusion, these results demonstrated that *Bacillus subtilis* and *Bacillus velezensis* supplementation can increase overall growth performance and ameliorate the blood parameters related to inflammation and immunity of mice.

**Electronic supplementary material:**

The online version of this article (10.1186/s12934-019-1161-6) contains supplementary material, which is available to authorized users.

## Background

It is well known that antibiotics play a crucial role in the treatment of bacterial diseases and the growth performance of animals [1]. However, numerous studies showed that confined animal feeding use antibiotics extensively and this induced the rise of antibiotic-resistant bacteria and dysbacteriosis in the animals [2, 3]. The symbiotic bacteria could display antibiotic resistant phenotype under frequent antibiotic exposure and transfer to humans in the end. Moreover, the indiscriminately use of antibiotics in the livestock causes a great threat to the health status of humans and food safety via food supply chain [4]. Previous research has shown that a large number of problems have emerged in animal production with the prohibition of antibiotics in some countries, such as reduced growth performance and outbreak of diseases which have been originally controlled by antibiotics [5]. Therefore, a new class of useful antibiotic alternatives was expected to be developed. To date, probiotics have been considered as natural substitutes of antibiotics due to their multiple beneficial effects on the host and have attracted extensive attention between the researchers [6].

Probiotics are live microorganisms, which could benefit the host when they colonized in the body [7]. In general, some lactic acid bacteria (*Lactobacillus johnsonii* and *Lactobacillus reuteri*) and few non-lactic acid bacteria (*Bacillus subtilis* and *Bacillus licheniformis*) were deemed as probiotics [8]. The main mechanism by which probiotics play a beneficial role in animals is described as follows: (i) production of antibiotic substances; (ii) compete with harmful bacteria for nutrients; (iii) adhesion and colonization in the intestinal mucosa; (iv) reducing stress response and enhancing immunity dint; (v) regulating the binding of cytokines to receptors to regulate immune response [9]. Several healthy and beneficial characteristics appeared when probiotics were administered to animals. Studies demonstrated that *Lactobacillus johnsonii* BS15 (CCTCC M2013663) could accelerate the growth performance of chicken and prevent nonalcoholic fatty liver disease in obese mice [10]. Some researchers have reported that *Clostridium butyricum* have a great effect upon the digestive enzyme activity, antioxidative capacity, immunizing power, intestinal mucosa [11]. Wang et al. have reported that the appropriate supplementation of *Bacillus subtilis* in broiler chickens diet could reduce the inflammatory response and heat stress-related behaviors [12].

Yaks are ancient species of the high-altitude environment characterized by adapting low temperature and hypoxic conditions. According to the statistics, 90% of the yaks in the world mainly inhabit Sichuan, Tibet, and Qinghai provinces of China [13]. Yaks could make full use of herbage resources in alpine areas and have strong adaptability to their harsh ecological environment conditions. Therefore, altitude hypoxia (low pressure and low oxygen) would exert significant pressure on evolutionary selection [14, 15]. Previous study has shown a significant difference in gut microbial communities between Tibetan living at high altitudes and Chinese Han living at lower mainland due to distinctive life environments and dietary habits [14]. Brigitta and Adak research showed that the composition of gut microbiota of human being may alter, when they were exposed to high-altitude in a short time [16, 17]. This indicated that yaks may also have a special gut bacteria compared to the animals living in plain region. However, there are few reports on the intestinal microorganisms of yaks, and even fewer studies on the probiotics in yaks.

In the present study, we aimed to assess the effects of two strains of *Bacillus subtilis* (named BS1 and BS2) and one strain of *Bacillus velezensisis* (named BV1) isolated from yaks on growth performance, organ index, small intestinal mucosa, intestine digestive capacity, antioxidant capacity, and immune indices of mice.

## Methods

### Probiotics culture

Two *Bacillus subtilis* strains (BS1 and BS2) and one *Bacillus velezensis* strain (BV1) were isolated from the intestines of yaks and were conserved in Laboratory of Veterinary Internal Medicine in Huazhong Agricultural University, Wuhan, China. Our previous study has demonstrated that BS1, BS2, and BV1 had high antibacterial efficacy against *Staphylococcus aureus* (ATCC 26112), *Escherichia coli* (ATCC 25922), and *Salmonella enteritidis* (NCTC 13349) and exhibited a high tolerance to acid and bile salts [18]. In addition, antibiotic resistance experiments, resistance gene tests, and hemolytic experiments were performed to determine the safety of the BS1, BS2, and BV1 strains. Our previous results showed that BS1, BS2, and BV1 had lower antibiotic resistance and resistance genes and hemolysis were not found [18]. The safety assessment results showed that the isolated strains were safe and could be used in animal experiments [18]. Three isolated strains were cultured in Luria–Bertani (LB) broth at 37 °C for 24 h under aerobic environment. Afterwards, the viable cells number of BS1, BS2 and BV1 were assessed by using plate count method. The bacterial suspensions were gathered under sterile conditions for oral administration in the mice.

### Animal experiment

Fifteen-day-old healthy Kunming mice (n = 72) were purchased from animal experiment center of Hubei Province at Wuhan, China. Mice were randomly divided into the control group, BS1-treated group, BS2-treated group, and BV1-treated group. During the whole experiment, mice were allowed drink and feed freely. In addition, mice in the probiotic-treated groups were gavaged BS1, BS2, or BV1 at 1 × 10^9^ CFU/day for 21 consecutive days and the control mice were received the same volume of saline with the same method. In each group, 18 mice were equally divided into 3 replicates. All the mice (15–18 g) had similar initial weights and nutrient contents of the diets. The mice were raised in plastic cages (size, 375 mm × 273 mm × 165 mm) for 21 days. During the experiment, the brooding temperature was maintained from 20 to 24 °C, the humidity was maintained from 53 to 57% and the daily lighting was performed as 12 h for light and 12 h for dark. In addition, three times a day (every 8 h), their diet, drinking water, and overall performance were recorded, death, diarrhea, depression, tiredness, loss of appetite was considered unnormal performance for the mice.

### Samples collection and processing

After 21 days of feeding, all groups’ mice were euthanized by injection of pentobarbital (25 mg/kg). Afterwards, the liver, kidney, intestines (duodenum, jejunum, and ileum) and blood samples were collected immediately under sterile conditions for further study. At the same time, mice were dissected by a surgical knife to observe internal organs and intestinal samples. The part of the collected intestinal samples (duodenum, jejunum, and ileum) approximately 2 cm, while liver, kidney, and spleen cut into cubes (0.5 cm × 0.5 cm × 0.5 cm) were fixed in 4% paraformaldehyde. On the other hand, all the fixed samples were routinely processed by embedding in paraffin and histological sections (4–5 µm) were stained with hematoxylin and eosin (H&E) for microscopic examination. The rest of the samples were stored at − 80 °C for further analysis. The blood samples were placed statically in the centrifuge tube for 1 h. Then, the samples were centrifuged at 3500×*g* for 10 min at 4° high-speed centrifuge (H2050R-1, Changsha, China) and the supernatants were stored at − 80 °C for ELISA and biochemical analysis.

### Production performance analysis

In this study, mice were weighted at fixed times and places. In addition, average daily feed intake (ADFI) and average daily weight gain (ADWG) were recorded and calculated by using each group weighted. The feed conversion ratio (FCR) was calculated with the following formula: $FCR = \frac{{{\text{ADFI}}}}{{{\text{ADG}}}}$.

### Morphological analysis of small intestinal mucosa

The morphology of small intestine histological sections stained with hematoxylin and eosin (H&E) were observed by invert microscope (Olympus BX51, Japan) and the length and depth of intestinal villus were measured by the image analysis program (Image-Pro Plus 6.1 Media Cybernetics, Rockville, MD, USA). Moreover, the ratio of villus length to crypt depth was also calculated.

### Digestive enzyme activity, antioxidant indexes and biochemical indexes

The liver and small intestine were homogenized by adding a sterile 0.9% saline solution to prepare 10% (w/v) homogenates and the homogenates were stored at − 80 °C for further study. Antioxidant indexes in the liver including T-AOC, MDA, GSH-PX, and SOD and digestive enzyme activity in the small intestine including α-amylase and trypsin were measured by using commercial kits of Nanjing Jiancheng Bioengineering Institute (Nanjing, China). Although probiotics supplementation has a beneficial effect on the host, but some probiotics may lead to liver damage and bacteremia [19]. Therefore, to evaluate whether probiotics isolated from Tibetan yaks induced liver damage in mice, liver indexes analysis was performed. In addition, biochemical indexes related to the liver (AKP, AST, and ALT) and serum (TP, GLB, ALB, Ca, P) were also performed by the commercial kits, which were purchased in the Nanjing Jiancheng Bioengineering Institute (Nanjing, China). All the operations are performed under the manufacturer’s protocols.

### Immunoglobulins and cytokines analysis

The serum immunoglobulins including IgG, IgM, and IgA levels and cytokines including IL-6, IL-8, IL-10, and TNF-α content were quantified using mice-specific ELISA kit (Colorful Gene Biological Technology, Wuhan, China) following manufacturer’s instructions. The OD (optical density) values of each sample were measured by the Thermo Scientific Microplate Reader in the 400 nm wavelengths.

### Statistical analysis

The data in this study were analyzed via χ^2^ test and student *t* test and expressed as mean ± SD. The SPSS statistical program was used to analyze all the data and P < 0.05 was considered statistically significant.

## Results

### Organ indexes analysis

**Fig. 1:**
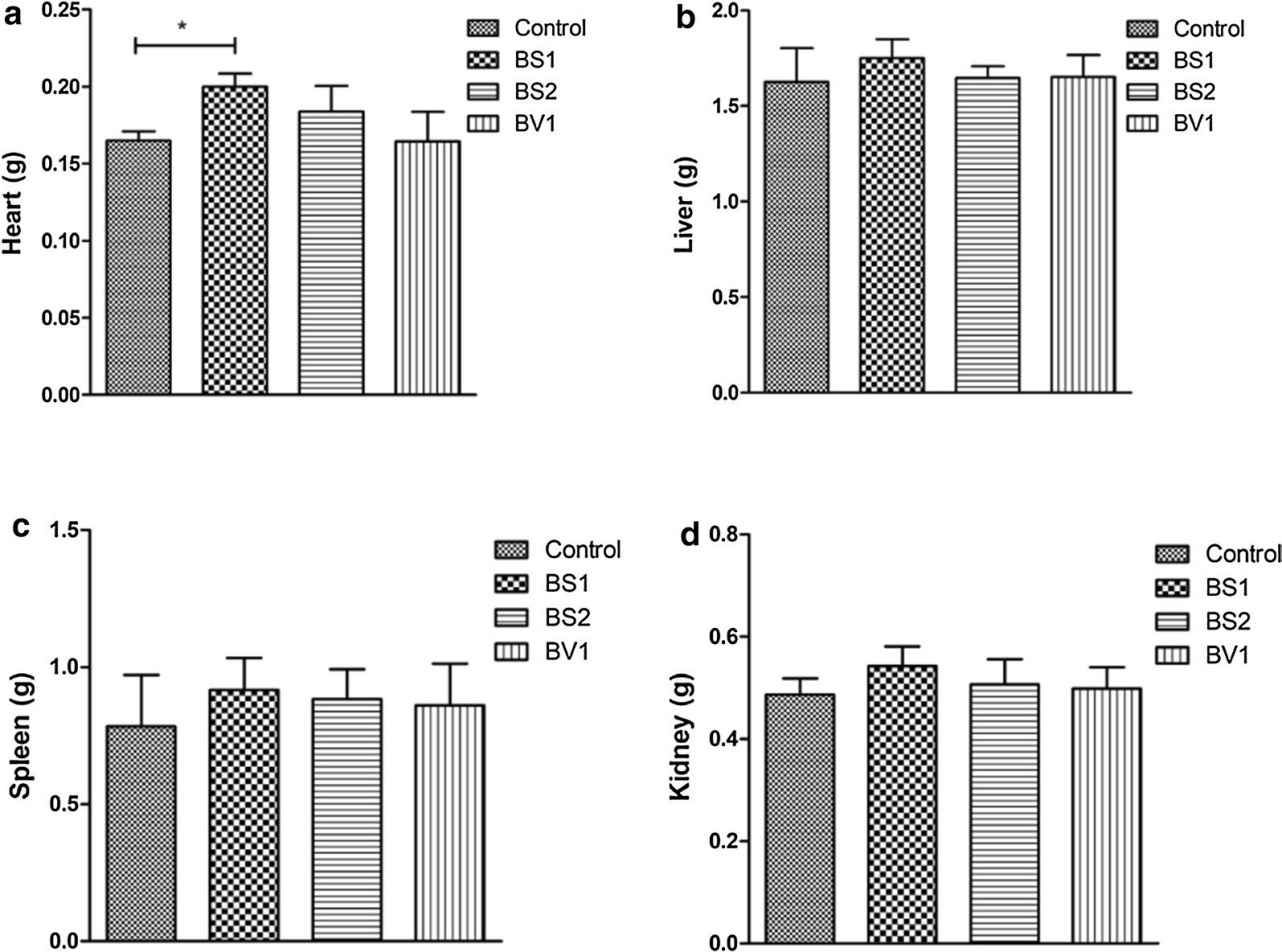
Heart, liver, kidney, and spleen indexes analysis in all the groups. **a** Heart weight; **b** liver weight; **c** spleen weight; **d** kidney weight. The data are expressed as the mean ± SD. *P < 0.05

During the experimental period, no any abnormal behaviors were observed in the experimental group compared with the control group. Moreover, no macroscopic alteration was found by autopsy and microscopic examination (Additional file 1: Fig. S1). All the collected heart, liver, kidney and spleen were weighed via electronic balance (JE1002, Puchun metrical instrument company, Shanghai, China). The results showed that there was no significant difference in the weight of liver, kidney, and spleen between the control group and probiotic-treated groups (P > 0.05) (Fig. 1b–d). However, mice supplemented with BS1 had a heavier heart compared with control group (P < 0.05) (Fig. 1a).

### Effects of probiotics administration on production performance

**Fig. 2:**
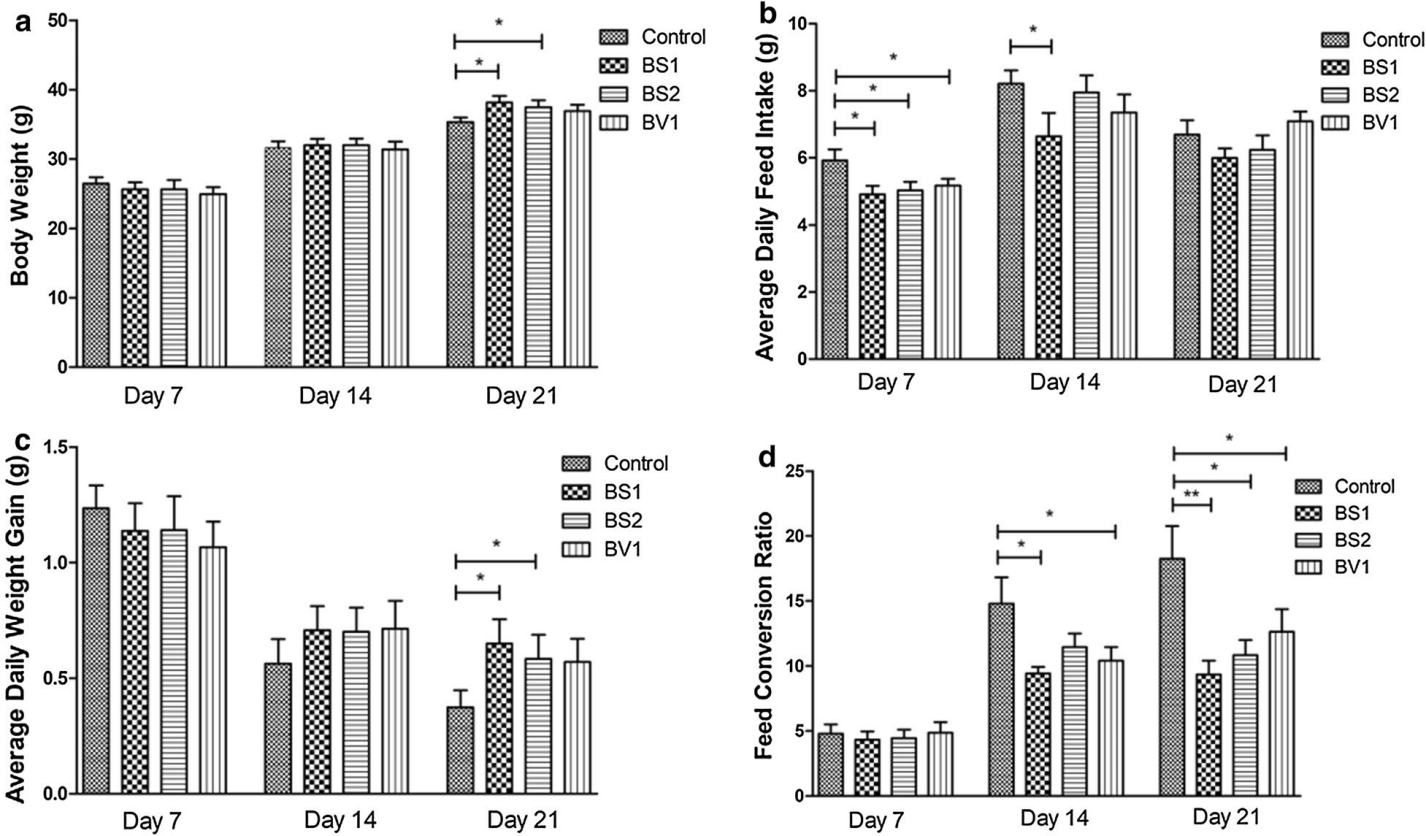
Probiotics supplementation increased the overall performance of experimental mice. **a** Body weight; **b** average daily food intake; **c** average daily weight gain; **d** the feed conversion ratio. Values indicate mean ± SD. *P < 0.05, **P < 0.01

Promoting animal production performance and reducing feed conversion rate are considered one of the characteristics of probiotics. In this study, probiotics supplementation enhanced the overall performance of the experimental mice. As shown in Fig. 2a, there was no difference in the body weight (BW) on day 7 and day 14 between the control group and experimental group. However, the weight of the mice treated with BS1 and BS2 was significantly higher than that of the control group on day 21 (P < 0.05) (Fig. 2a). The average daily food intake (ADFI) in the probiotic-treated mice was lower than the control group at the start of the study (day 1 to day 7) (P < 0.05) (Fig. 2b). BS1, BS2, and BV1 treated mice showed a commendable growth trend from 7 days, and the average daily weight gain (ADWG) of mice treated with BS1 and BS2 was significant difference with the control mice on day 14 to day 21 (P < 0.05) (Fig. 2c). Moreover, the feed conversion ratio (FCR) was markedly reduced in the probiotic-treated groups compared with the control group on day 21 (Fig. 2d) (P < 0.05 or P < 0.01). The above results indicated that BS1 and BS2 supplementation improved the growth of mice.

### Probiotics supplementation improve the small intestinal mucosa morphology

**Fig. 3:**
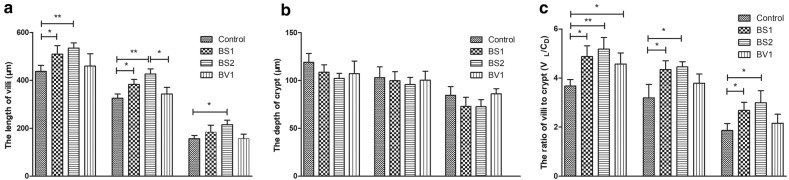
Probiotics supplementation improved the overall histomorphometric parameter of the duodenum, jejunum, ileum. **a** The length of villus in the small intestine; **b** the depth of crypt in the small intestine; C: the ratio of villus length to crypt depth. The data are expressed as the mean ± SD. *P < 0.05, **P < 0.01

Under normal circumstances, the absorbing ability is determined by the morphology of the small intestinal mucosa. Therefore, to evaluate the changes in the length of intestinal villi and the depth of crypt under probiotics supplementation conditions, morphology of small intestinal mucosa analysis was performed. During the statistical analysis, we noticed that the length of villi in the duodenum and jejunum of the BS1- and BS2-treated groups was increased compared with control group (P < 0.05) (Fig. 3a). Similarity, the length of villi in the ileum was elongated in mice treated with BS2 compared with control group (Fig. 3a). As shown in Fig. 3b, no significant difference in the crypt depth of small intestine was observed between the probiotic-treated groups and the control group. In addition, supplemented with BS1, and BS2 significantly increased the ratio of V/C (villus length/crypt depth) in the duodenum, jejunum, and ileum compared with the control group (P < 0.05 or P < 0.01) (Fig. 3c). However, similar results observed only in the duodenum of the BV1-treated group (P < 0.05) (Fig. 3c). All the data identified that probiotics supplementation significantly improve the morphology of small intestinal mucosa.

### Digestive enzyme activity analysis

**Fig. 4:**
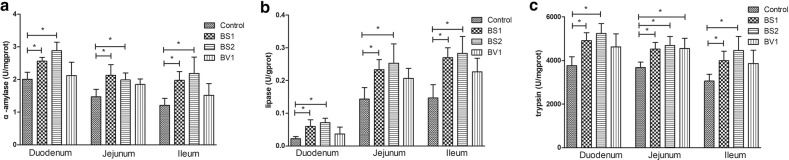
Probiotics supplementation improved the digestive enzyme activity of duodenum, jejunum, and ileum. **a**–**c** The levels of α-amylase, lipase, and trypsin in the small intestine. The data are expressed as the mean ± SD. *P < 0.05, **P < 0.01

It is well known that the decomposition and absorption of food are inseparable from digestive enzyme. Research indicated that the lack of digestive enzyme can lead to indigestion syndrome and affect the growth of animals. Therefore, in order to evaluate the effect of *Bacillus subtilis* and *Bacillus velezensis* isolated from Tibetan yaks on the activity of digestive enzyme in mice, the levels of α-amylase, lipase, and trypsin in small intestine (duodenum, jejunum, and ileum) were detected. The digestive enzyme indices in control and probiotic-treated mice were exhibited in Fig. 4a–c. The results of our present studies showed that probiotics supplementation can improve the digestive enzyme activity of small intestine. Specifically, α-amylase, lipase, and trypsin levels in small intestine were significantly increased after feeding BS1 and BS2 (Fig. 4a–c) (P < 0.05). Although, BV1-treated group improved the activity of related digestive enzyme, the significant difference was observed only in trypsin of jejunum (Fig. 4c) (P < 0.05).

### Probiotics supplementation improves the antioxidant ability of mice

**Fig. 5:**
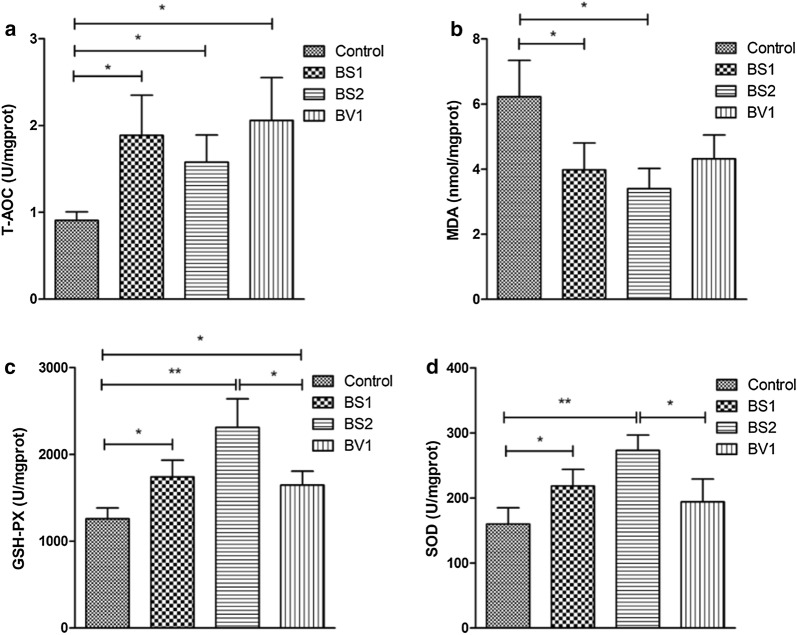
Probiotics supplementation improved the antioxidant capacity of mice. **a** The total antioxidation capacity (T-AOC) level of liver in each group; **b** the maleic dialdehyde (MDA) of liver in each group; **c** the glutathione peroxidase (GSH-PX) of livers in each group; **d** the superoxide dismutase (SOD) of livers in each group. The data are expressed as the mean ± SD. *P < 0.05, **P < 0.01

The liver antioxidant indices in control and probiotic-treated mice were shown in Fig. 5a–d. The results showed that mice treated with BS1 and BS2 exhibited significantly higher T-AOC and GSH-PX levels (Fig. 5a, c) (P < 0.05 or P < 0.01) but significantly lower MDA contents (Fig. 5b) (P < 0.05). Compared with control mice, BV1-treated mice strengthened the activity of T-AOC and GSH-PX (Fig. 5a, c) (P < 0.05). However, no significant difference in MDA and SOD levels was observed (Fig. 5b, d) (P > 0.05). Furthermore, significant changes could be observed in GSH-PX and SOD levels between BS2- and BV1-treated groups (Fig. 5c, d) (P < 0.05).

### Biochemical indices analysis

**Fig. 6:**
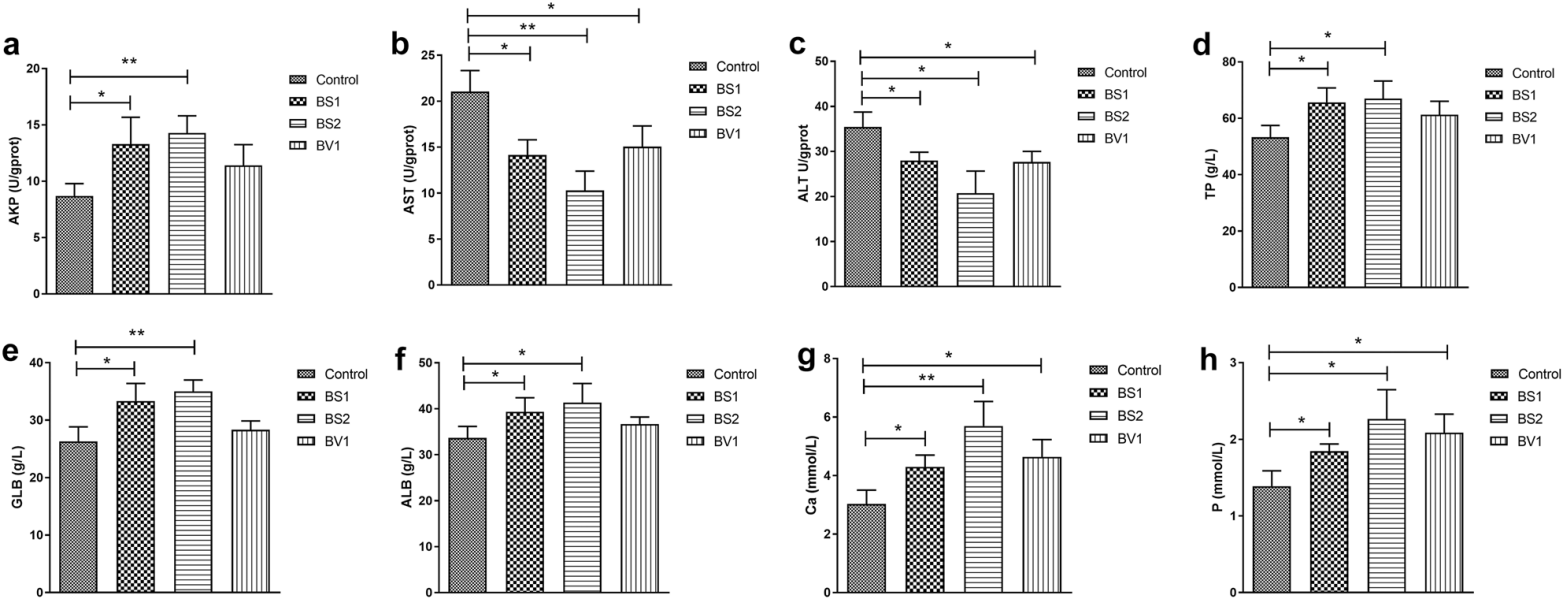
Effects of probiotics supplementation on biochemical index in mice. Effects of probiotics on biochemical index in mice. **a**–**c** AKP, AST, and ALT contents in the liver of each group; **c**–**h** TP, GLB, ALB, Ca, P contents in serum of each group. The data are expressed as the mean ± SD. *P < 0.05, **P < 0.01

As shown in Fig. 6a, the content of AKP in the BS1- and BS2-treated groups was significantly increased as compared with control group (P < 0.05 or P < 0.01). Additionally, statistical analysis showed that AST and ALT levels in the experimental mice were lower than the controls (P < 0.05 or P < 0.01) (Fig. 6b, c). At the same time, the levels of Ca, P, TP, GLB, and ALB in the serum were also analyzed. The contents of serum Ca and P in the experimental group were markedly increased as compared to control group (P < 0.05 or P < 0.01) (Fig. 6f, g). Furthermore, mice supplemented with probiotics exhibited higher serum TP, GLB, and ALB contents than the controls, except the BV1-treated mice (P < 0.05 or P < 0.01) (Fig. 6d–f).

### Levels of immunoglobulin

**Fig. 7:**
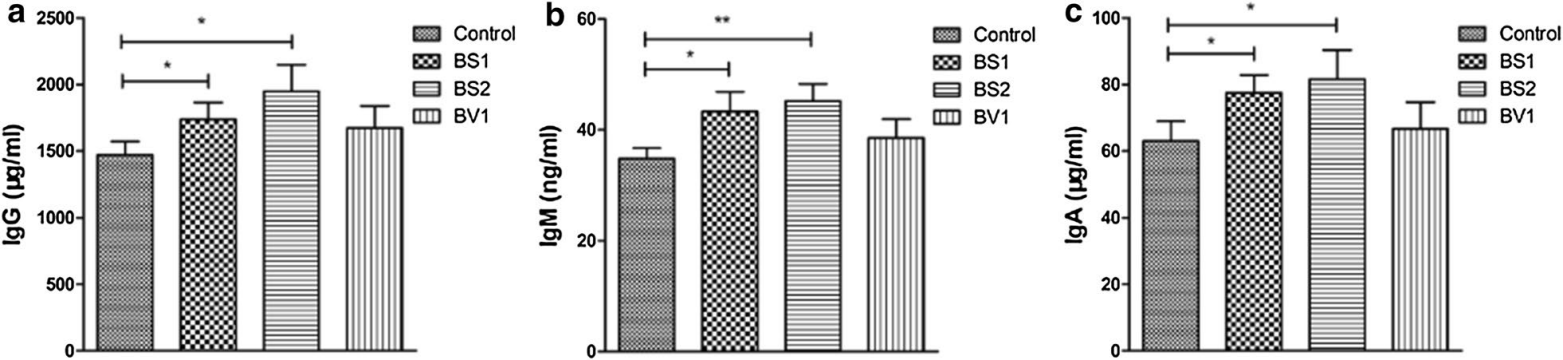
Effects of probiotics supplementation on the serum-associated immune factors in mice. **a**–**c** Serum IgG, IgM and IgA levels in the different groups. Data are expressed as the mean ± SD. *P < 0.05, **P < 0.01

Serum immunoglobulin levels of mice were summarized in Fig. 7 and the concentration of immunoglobulin in mice showed varying degrees of changes after supplementation with probiotics. As shown in Fig. 7, BS1 and BS2 supplement significantly improved serum IgG, IgM, and IgA levels in mice as compared to controls (P < 0.05). Whereas, no significant difference (P > 0.05) was observed between the control group and BV1-treated group (Fig. 7).

### Cytokines levels

**Fig. 8:**
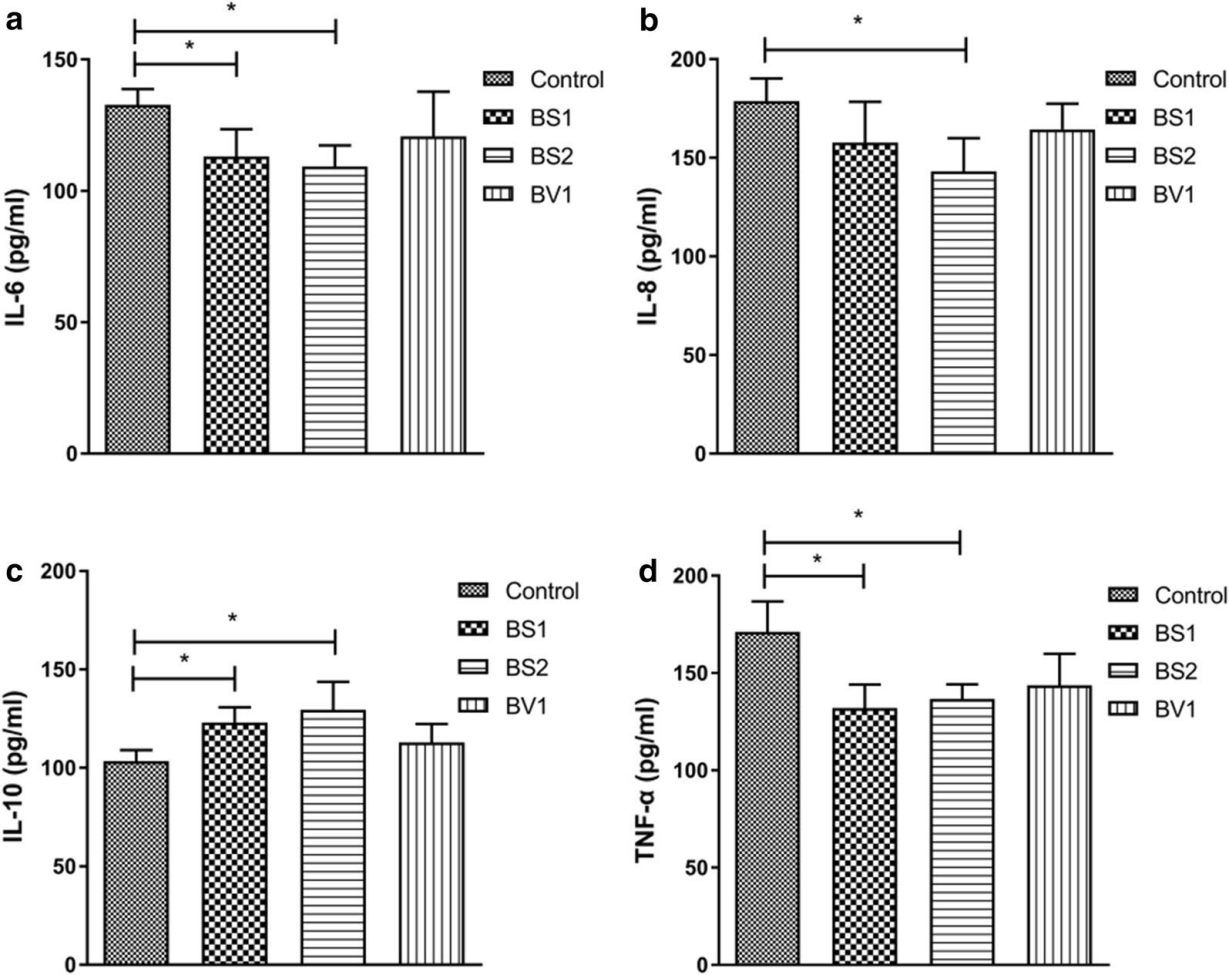
Effects of probiotics supplementation on the serum-associated inflammatory agents in mice. **a**–**d** Serum IL-6, IL-8, IL-10, and TNF-α level in the different groups. Data are expressed as the mean ± SD. *P < 0.05, **P < 0.01

During the statistical analysis of data, we observed that the experimental group had higher IL-10 levels than the control group, especially the group treated with BS1 and BS2 (P < 0.05) (Fig. 8). In addition, Fig. 8a, d demonstrated that the levels of IL-6 and TNF-α were decreased, and significant difference was noted after mice given BS1 and BS2 compared with the control mice (P < 0.05). Figure 8c reveals the content of IL-8 in the serum of mice in different groups. The significant (P < 0.05) changes were observed only between the control group and BS2-treated group.

## Discussion

At present, large quantities of antibiotics were still used as feed additive to promote growth performance [20]. However, heavy use of antibiotics may lead to dysbacteriosis, drug resistance, and even antibiotic associated diarrhea (AAD) [21]. Increasing evidence indicated that probiotics supplementation could promote animal growth and inhibit the pathogenic microorganism [11, 22]. Hypoxia and hypotension may alter the gut microbes of yaks. On the other hand, yaks could make full use of herbage resources in alpine areas and have strong adaptability to their harsh ecological environment conditions. Therefore, microorganisms in the intestines of yaks may also have these characteristics. Yaks are generally raised free-range and rarely given antibiotics. Additionally, the ecological environment of the Tibetan plateau is very good and neither the forage grass nor the water source has been polluted. Not only that, forages is generally free growing and rarely uses pesticides. Therefore, probiotics with lower antibiotic resistance may be isolated from the intestines of yaks. In the present study, *Bacillus subtilis* and *Bacillus velezensis* were isolated from the intestines of yaks and mice were used as a model to test the possible probiotics proprieties. In this study, we firstly demonstrated that probiotics isolated from Tibetan yaks can improve the growth performance of mice.

The bulk of nutrition absorption occurs in the small intestine and positively correlated with the small intestinal villi length. Previous studies have shown that probiotics supplementation could increase the length and lower the depth of crypt of intestinal villi [11]. In the present study, the morphology of the small intestine was analyzed. The results showed that the length of villus and the ratio of the villus length to crypt depth were increased and the depth of crypt was reduced in the small intestine after in taking probiotics. The digestive function of animals depends on the mechanical digestion of gastrointestinal movement and the chemical digestion of digestive enzymes [23, 24]. Digestive enzymes are mainly stored in digestive juice, which can promote the hydrolysis of sugar, fat, and protein in food, and convert macromolecular matter into small molecules that can be absorbed and utilized to the organism. In this study, the relevant digestive enzymes in the small intestine were analyzed including α-amylase, trypsin, and lipase and the data showed that the digestive enzymes in the duodenum, jejunum, and ileum were heightened. More specifically, the levels of α-amylase, trypsin, and lipase in small intestine increased by 28.54%, 13.16%, and 71.43% in BS1-treated group and 43.28%, 26.32%, and 76.92% in BS2-treated group, respectively. It is known that the growth and development of animals cannot separate from the absorption of nutrients. It also means that intestinal villi and digestive enzymes are indispensable for the growth of animals. In the present study, the BW, ADWG, and FCR have significantly improved in the BS1- and BS2-treated groups on day 21. However, no significant difference of ADFI was observed between control and probiotic-treated groups. Mice supplemented with BS1 and BS2 have higher final body weight without increasing food intake, which means that the probiotic-treated mice possess better absorption and digestion capabilities. Previous studies has shown that the increase in the length of the intestine villi contributes to provide a vast absorptive surface area which is considered to be a key factor in promoting growth performance [25]. In addition, higher digestive enzyme activity can make food digestion more thorough, which is also more conducive to the absorption of the small intestine. In the present study, our results indicated that probiotics supplementation could significantly improve overall daily weight gain of mice and increased the length of intestinal villi and digestive enzyme activity. Our results were consistent with previous studies that probiotics can promote animal growth by improving small intestinal mucosa morphology and digestive enzyme activity [26].

There were reports that oxidative stress associated with kidney disease and may lead to molecular lesions and trigger apoptosis [27, 28]. Furthermore, Chiva’s research showed that intestinal mucosal oxidative damage may induce bacterial translocation [29]. Therefore, antioxidant ability is vital for health of animals. T-AOC, GSH-Px, SOD, and MDA are important indicators for evaluating the antioxidant capacity of the liver. The results in this study suggested that the levels of those antioxidant indices in the liver were positively influenced after probiotics supplementation. MDA is one of the end products of membrane lipid peroxidation and its content can be used as one of the indicators to assess the severity of stress in cells [30]. The main damage of MDA production is to damage the structure of biological membrane, altering the permeability of membrane, and increases membrane fragility, thus affecting the normal progress of a series of physiological and biochemical reactions [31]. The SOD could eliminate the harmful substances produced in the metabolic process of organisms, which is called the first-line cellular defense against oxidative damage [32]. More importantly, SOD plays a primary role in anti-aging and catalytic peroxide anion [33]. Aside from the above-mentioned antioxidant indexes, GSH-Px also play a vital role in preventing cellular oxidative damage via decomposes hydrogen peroxide [34]. Environmental stress can accelerate the production of reactive oxidative species (ROS) and lead to oxidative stress [35]. Oxidative stress can not only induce several diseases but also affect the growth rate, meat quality and feed conversion rate of animals [36, 37]. Meanwhile, previous study has shown that antioxidant enzyme can reduce ROS production and prevent oxidative stress [38]. Additionally, antioxidant enzyme can also repair oxidant damage induced by oxidative stress [38]. Therefore, improving the activity of antioxidant enzyme is very important for the healthy growth and development of animals. Previous research has shown that probiotics supplementation can increase the levels of antioxidant enzyme, which was considered an important signal to improve antioxidant capacity [39]. In the present study, the levels of MDA was reduced and the levels of T-AOC, SOD, GSH-Px were increased in the BS1- and BS2-treated groups, which suggested that probiotics supplementation boost the antioxidant ability of mice. So, our study demonstrated that probiotics can be used as an effective additive to improve the antioxidant capacity of animals [40].

AST and ALT are crucial aminotransferases in the animal body, which are considered as a valuable indicator of hepatic injury [41]. In the general case, AST and ALT can maintain a dynamic balance without significant changes. However, the levels of AST and ALT will significantly increase, when the pathological damage appeared in the liver [42]. Previous studies have shown that liver damage induced by CCl_4_ lead to a significant increase in the levels of ALT and AST [43]. Previous research showed that dietary supplementation with *Bacillus subtilis* could reduce hepatic injury in broilers [44]. Previous research had revealed that AKP is an extracellular enzyme, which plays a significant role in immune defense, proteolysis [45, 46]. In our study, AST and ALT levels in the probiotic-treated groups was significantly lower than the control group, which suggested that *Bacillus subtilis* isolated from Tibetan yaks can alleviate the liver injury of mice to some extent. In addition, the levels of AKP in the experimental group also increased compared to the control group. Ca and P are important mineral elements in the body and play a vital role in the calcification of bones and teeth [47]. The rapid growth of animals requires the consumption of large amounts of calcium and phosphorus to support bone formation. Several bone tissue diseases and growth problems are caused by Ca and P deficiency. Such as, lack of Ca and P in young stock may induce rickets [48]. The fast-growing broilers are prone to tibial dyschondroplasia due to calcium and phosphorus deficiency [49]. ALB and GLB are the most important proteins in blood, which play a vital role in transporting metabolites, nutrition, and maintaining colloid osmotic pressure. Moreover, GLB is also called immunoglobulin, and could reflect the immunity of animals. We found that the *Bacillus subtilis* strains isolated from Tibetan yaks significantly increased the levels of Ca, P, TP, ALB, and GLB, suggesting that probiotics provide nutrients for the animal growth. Notably, probiotic-treated groups exhibited higher levels of GLB, indicating that probiotics supplementation may improve immune performance in mice.

Previous research has shown that immunoglobulins play an important role in immune regulation and mucosal defense [50]. IgG is the highest serum immunoglobulin and can reflect the systemic immune status of animals. IgM is the earliest antibody synthesized and secreted during ontogenesis and is also the earliest antibody to appear in the initial humoral immune response. Simultaneously, IgM can also accelerate the production of IgG [51]. IgA can be divided into serotype IgA and secretary IgA (sIgA) according to the immune function. IgA is the main antibody of mucosal local immunity, which can form a biological barrier to prevent and neutralize exogenous invading pathogen from colonizing in mucosal surface [52]. Beyond that, serum IgA mediates some protective functions through interaction with specific receptors and immune mediators. Recently it is demonstrated that, *Bacillus subtilis*, *Bifidobacterium lactis*, and *Bifidobacterium infantis* contribute to enhance the levels of IgA, IgM, and IgG in the host serum [53–55]. In this study, we found that probiotics supplementation increased serum IgG, IgM, and IgA levels of the experimental mice compared with the control mice, which indicated that *Bacillus subtilis* and *Bacillus velezensis* isolated from Tibetan yaks may modulate the immunity in mice to a certain degree.

As acknowledged inflammatory suppressor, IL-10 plays an important role in conditioning the intestinal homeostasis, inhibiting the release of inflammatory mediators, promoting natural and specific immunity [56, 57]. Previous research has shown that IL-10 deficiency may induce intestinal inflammation and chronic intestinal inflammation [58]. IL-6, IL-8 and TNF-α are the most important pro-inflammatory cytokines, which plays a key role in the development of inflammation. Helper T (Th) cells are a type of T cells, which can act as helper or inflammatory T cells in response to exogenous antigens [59]. Th cells can be divided into two main classes. One type of the Th cells can activate other T cells to achieve cellular inflammatory responses, and the other can promote humoral immune response [60]. Some researchers suggested that IL-6 can regulate the activity of pathogenic Th cells to amplify and perpetuate chronic inflammation [61]. Furthermore, the expression of TNF-α was increased in inflammatory mucosal tissues [62]. Persistent colonization of *Helicobacter pylori* in the stomach may lead to gastroduodenal inflammation and digestive ulcer and induces the production of IL-8 [63, 64]. Some research data showed that IL-8 is an effective neutrophil-activating and chemotactic factor and plays a leading role in inflammatory response caused by *Helicobacter pylori* [65, 66]. Over expression of cytokines may cause systemic inflammation out of control and induced infectious shock and necrotic enterocolitis [67]. Increasing evidences showed that probiotics colonization in the intestines contributed to form the anti-inflammatory circumstance and reduced the production of relevant pro-inflammatory cytokines. Such as, *Bacillus subtilis* PB6 can reduce the levels of IL-6 and TNF-α in the serum, thereby relieving the colon mucosal inflammation [68]. Furthermore, it has been proved that probiotic *Clostridium butyricum* supplementation increased the levels of IL-10 to alleviate the experimental colitis in mice [69]. Similar results were also observed in our present study that probiotics supplementation reduced the levels of IL-6, IL-8 and increased IL-10 levels. Such results correspond with the previous studies, suggested that probiotics isolated from Tibetan yaks exerted protective and beneficial effects in mice via controlling cytokines secretion. The infectious diseases can reduce the productivity of animals all over the world [70–73].

Before performing the animal experiment, some in vitro tests were performed to evaluate the antibacterial activity and safety of BS1, BS2 and BV1, including acid and bile salts tolerance tests, hemolytic activity tests, antibacterial tests, antibiotic susceptibility assay, and resistance gene tests. The results showed that BS1, BS2, and BV1 had the potential as probiotics [18]. The results of animal experiment demonstrate that the *Bacillus subtilis* isolated from Tibetan yaks can increase the growth performance of mice by improving digestive enzyme activity and the length of intestinal villi. More importantly, mice treated with BS1 and BS2 exhibited superior properties on antioxidant ability and cytokines related to immunity and inflammation. In summary, our study suggested that BS1 and BS2 isolated from Tibetan yaks can be used as a safe and effective probiotics additive. In addition, our studies also support the increasing scientific evidence that probiotics supplementation may have a healthy and beneficial effect on the host.

## Additional file

**Additional file 1: Fig S1.** Histological examination in the liver (A1–A4), kidney (B1–B4), and spleen (C1–C4).

## Data Availability

It will be available on request.
