# Supplementary figures and images for: Probiotics isolated from yaks improves the growth performance, antioxidant activity, and cytokines related to immunity and inflammation in mice
Source: Microb Cell Fact. 2019 Jun 19;18:112. doi: 10.1186/s12934-019-1161-6 (PMC6585042; doi:10.1186/s12934-019-1161-6)

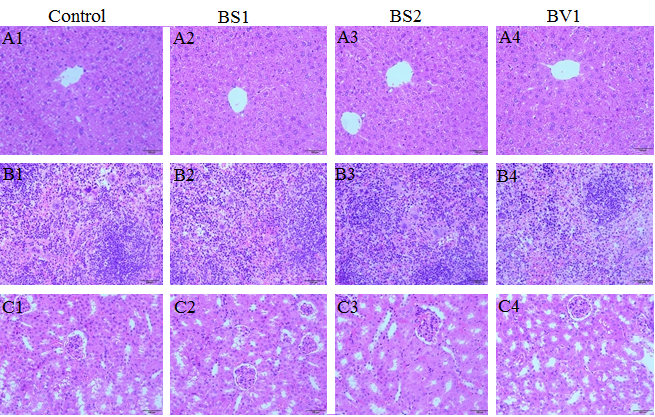


Fig. S1. Histological examination in the liver (A1-A4), kidney (B1-B4), and spleen (C1-C4)

Supplement: Supplementary file 1 — Additional file 1: Fig S1. Histological examination in the liver (A1–A4), kidney (B1–B4), and spleen (C1–C4). [file 12934_2019_1161_MOESM1_ESM.docx]
